# Retraction Note: *ω*-3 free fatty acids and all-trans retinoic acid synergistically induce growth inhibition of three subtypes of breast cancer cell lines

**DOI:** 10.1038/s41598-024-77048-8

**Published:** 2024-11-05

**Authors:** Guangxiao Lin, Shenglong Zhu, Yikuan Wu, Ci Song, Wanjing Wang, Yuan Zhang, Yue-Lei Chen, Zhao He

**Affiliations:** 1 State Key Laboratory of Food Science and Technology, School of Food Science and Technology, Jiangnan University, Wuxi, 214122 China; 2 Synergistic Innovation Center for Food Safety and Nutrition, Jiangnan University, Wuxi, 214122 China; 3 School of Medicine, Jiangnan University, Wuxi, 214122 China; 4 Institute of Biochemistry and Cell Biology, Shanghai Institutes for Biological Sciences, Chinese Academy of Sciences, Shanghai, 200031 P.R. China

Retraction of: *Scientific Reports* 10.1038/s41598-017-03231-9,  published online 02 July 2017

After publication of this Article, concerns about the data were reported to the Editors. Specifically, several images published in this Article appear to have also been included in another publication that was under review simultaneously to this Article with common authors [1], where the images are described differently. These include:

- DHA and A + RA images in MCF7 panel of Figure 1c;
- The B-actin blot of MDA-MB-23 panel in Figure 5e.

Additionally, the two β-Actin blots in Figure 4b of this Article appear highly similar to each other, even though these are presented for different experiments. The Authors were not able to provide raw data for this Article upon request by the Editors. The Editors therefore no longer have confidence in the reliability of the results and findings that it reports.

All authors disagree with this retraction.
